# Identification and functional characterization of a fish-specific *tlr19* in common carp (*Cyprinus carpio* L.) that recruits TRIF as an adaptor and induces *ifn* expression during the immune response

**DOI:** 10.1186/s13567-021-00957-3

**Published:** 2021-06-15

**Authors:** Shijuan Shan, Rongrong Liu, Hanxiao Feng, Fei Meng, Muhanmmad Aizaz, Guiwen Yang

**Affiliations:** Shandong Provincial Key Laboratory of Animal Resistance Biology, College of Life Sciences, Shandong Normal University, No.88 East Wenhua Road, Jinan, 250014 China

**Keywords:** *Cyprinus carpio* L., Toll-like receptor 19 (Tlr19), Innate immunity, Signaling pathway, *Ifn*

## Abstract

Toll-like receptor 19 (Tlr19) is a fish-specific TLR that plays a critical role in innate immunity. In the present study, we aimed to identify *tlr19* from common carp (*Cyprinus carpio* L.) and explored its expression profile, localization, adaptor, and signaling pathways. A novel *tlr19* cDNA sequence (*Cctlr19*) was identified in common carp. Phylogenetic analysis revealed that *Cc*Tlr19 was most closely related to *Danio rerio* Tlr19. Subcellular localization analysis indicates that *Cc*Tlr19 was synthesized in the free ribosome and then transported to early endosomes. *Cctlr19* was constitutively expressed in all the examined tissues, with the highest expression in the brain. After poly(I:C) and *Aeromonas hydrophila* injection, the expression of *Cctlr19* was significantly upregulated in immune-related organs. In addition, the expression of *Cctlr19* was upregulated in head kidney leukocytes (HKL) upon stimulation with different ligands. Immunofluorescence and luciferase analyses indicate that *Cc*Tlr19 recruited TRIF as an adaptor. Furthermore, *Cc*Tlr19 can activate the expression of *ifn-1* and *viperin*. Taken together, these findings lay the foundation for future research to investigate the mechanisms underlying fish *tlr19*.

**Supplementary Information:**

The online version contains supplementary material available at 10.1186/s13567-021-00957-3.

## Introduction

The innate immune system senses danger signals through a variety of germline-encoded pattern-recognition receptors (PRR) [1]. Toll-like receptors (TLRs) constitute a well-known family of PRR that are ubiquitously expressed in immune and nonimmune cells [2, 3] and link innate and adaptive immunity [4]. TLR are type-I transmembrane glycoproteins that are composed of three domains [5]: an extracellular leucine-rich repeat domain (LRR), a transmembrane domain (TM) and a cytoplasmic Toll/interleukin-1 receptor (TIR) domain [6]. The extracellular leucine-rich repeat domain of TLR recognizes bacterial and viral constituents, including lipids, lipoproteins, proteins and nucleic acids [7], while the intracellular Toll/interleukin-1 receptor (TIR) domain can recruit adaptors [8]. Upon stimulation with pathogen-associated molecular patterns (PAMP), the intracellular TIR domain recruits a series of adaptors and activates immune signaling cascades, including myeloid differentiation primary response 88 (MyD88)-dependent and MyD88-independent pathways [9]. The TLR-mediated signaling cascade induces transcription factors such as nuclear factor kappa-light chain-enhancer of activated B cells (NF-κB), mitogen-activated protein kinase (MAPK), activating protein-1 (AP-1) and interferon regulatory factor (IRF) family members, resulting in the production of inflammatory cytokines, chemokines, and/or antimicrobial peptides [10].

To date, at least 28 functional TLR have been identified in vertebrates, including at least 13 TLR in mammals and 22 TLR in fish [11]. Teleost fish TLR include mammalian TLR orthologs (TLR1, TLR2, TLR3, TLR5, TLR7, TLR8 and TLR9) and fish-specific TLR (soluble TLR5, TLR14, TLR18-20, and TLR22-28) [12]. TLR19 is a fish-specific TLR that has been reported in only limited fish species. It was first identified in zebrafish (*Danio rerio*) [13] and subsequently reported in channel catfish (*Ictalurus punctatus*) [14], Atlantic salmon (*Salmo salar*) [15], Tibet fish (*Gymnocypris przewalskii*) [16], grass carp (*Ctenopharyngodon idella*), bluntnose black bream (*Megalobrama amblycephala*) [17] and yellow catfish (*Pelteobagrus fulvidraco*) [18]. Previous studies have shown that basal expression of TLR19 is prevalent in immune tissues such as the spleen, head kidney and gill tissue [19], and its gene expression pattern can be modulated by various PAMP from various bacteria and viruses. In channel catfish, yellow catfish and rainbow trout, the expression of TLR19 was significantly upregulated following treatments with *Aeromonas hydrophila* or *Edwardsiella ictalurid* [14, 18]. Concerning viruses, TLR19 in grass carp and yellow catfish was also induced upon stimulation with poly(I:C) or grass carp reovirus (GCRV) [18, 20]. These results indicate that TLR19 plays a key role in innate immune responses in teleosts.

Common carp (*Cyprinus carpio* L*.*) is a freshwater fish that is widespread worldwide and accounts for as much as 10% of freshwater aquaculture production [21]. Since host TLR play important roles against pathogen responses, the study of TLR is beneficial for the disease defense of the common carp. To date, TLR1 [22], TLR2 [23], TLR3 [24], TLR5 [25], TLR9 [26], TLR18 [27], TLR20 [28] and TLR22 [29] have been reported in common carp. However, the functions and activating signaling pathways of *Cc*Tlr19 remain unknown. In the present study, we identified the expression patterns and preliminary function of the *Cc*Tlr19 gene after bacteria and poly(I:C) stimulation. Further studies found that *Cc*Tlr19 was synthesized in the free ribosome, did not reside in the endoplasmic reticulum, recruited TRIF and induced *ifn* expression. These findings will provide insight into the function of *Cc*Tlr19 in teleosts.

## Materials and methods

### Fish rearing and immune challenge

Common carp (*C. carpio* L*.*) with a body weight of approximately 180 g, were obtained from a local fish farm and raised in a laboratory at 25 °C for at least 1 week. Immune challenges were performed according to previously described methods [30]. Briefly, fish were injected intraperitoneally with formalin (0.5% formalin overnight at 4 °C), inactivated *Aeromonas hydrophila* (2 × 10^7^ CFU per fish) and poly(I:C) (1.6 mg/mL) at a dose of 500 μL. The control group was injected with the same amount of PBS. The samples were collected from three fish at different time points after stimulation (3 h, 6 h, 12 h, 24 h, 48 h, 72 h, 120 h and 168 h). The protocol was approved by the Animal Experimental Ethics Committee of Shandong Normal University (Permit Number: AEECSDNU2019038).

### Cell culture and transfection

293 T cells and HeLa cells were grown in DMEM (Gibco, USA) supplemented with 10% fetal bovine serum (Gibco), 100 U/mL penicillin and 100 μg/mL streptomycin (Gibco) and maintained at 37 °C in a 5% CO_2_ incubator. Epithelioma papulosum cyprinid (EPC) cells were maintained in M199 medium (Gibco) at 25 °C. Transfection was performed as previously described [27]. Lipofectamine 2000 (Invitrogen, USA) was used for 293 T cell transfection, FuGENE HD (Promega, USA) was used for HeLa cell transfection, and jetPRIME reagent (Polyplus, French) was used for EPC cell transfection according to the manufacturer’s instructions.

### Gene cloning and plasmid construction

To obtain the full-length cDNA sequence of *Cctlr19*, the partial sequence of *tlr19* was cloned from common carp using a pair of primers specific to the conserved region of the reported *tlr19* sequence. Then, 5′ and 3′ RACE-PCR was performed using a 3′-full RACE core set (Takara, China) and SMARTer^®^ RACE 5′ Kit (Clontech, USA) according to the manufacturer’s instructions.

For promoter analysis, 5′ flanking sequences of *ifn-1*, *ifn-2*, *ifn-3* and *ifn-γ* upstream of the first ATG were cloned into PGL4.10 basic plasmids with the indicated restriction enzymes. The generated recombinant plasmids were named Luci-*Ccifn-1*, Luci-*Ccifn-2*, Luci-*Ccifn-3* and Luci-*Ccifn-γ*. Eukaryotic expression vectors were made by insertion of the corresponding ORF into pEGFP-N1/pmCherry-N1/pFUGW with the indicated restriction enzymes. The primers used in this study are listed in Table 1.

**Table 1:** **Primer sequences used in this study**

Primer name	Primer sequence (5′–3′)	Usage
TLR19-F	TGCATCGATGCTGAGTCGCTG	*tlr*19 cloning
TLR19-R	GGCAGAGCTTCTCCATTGTGGCCA	*tlr*19 cloning
TLR19-5′outer	AGCGAGCAAAGCGATAACCAGCGG	5′RACE
TLR19-5′inner	GCGATAACCAGCGGCTGCCAACAG	5′RACE
TLR19-3′outer	GGAACAAGGGAACCCGAGACTGA	3′RACE
TLR19-3′inner	GAGAGTATCCACAGCAGTCAGTGC	3′RACE
TLR19-NI-F	ACATCCTGCAGGAATGGGTGTGCATGACTCC	Plasmid construction
TLR19-SI-R	CTAGCTAGCTCAAGAAGCTTCCGCGTC	Plasmid construction
TLR19-EI-F	CGGAATTCATGGGTGTGCATGACTCC	Plasmid construction
TLR19-SI-R	GGATTCCCTCAAGAAGCTTCCGCGTC	Plasmid construction
rtTLR19-F	GCCGCTGGTTATCGCTTTGCT	Real-time PCR
rtTLR19-R	ATCCTCCTGTGCCACTGCCTAC	Real-time PCR
rtS11-F	CCGTGGGTGACATCGTTACA	Real-time PCR
rtS11-R	TCAGGACATTGAACCTCACTGTCT	Real-time PCR
Rab5-F	CCCAAGCTTATGGCAGGAAGAGGCGGA	Plasmid construction
Rab5-R	CGGGGTACCGTGTTGCTACAGCGGGACC	Plasmid construction
Rab7-F	CCCAAGCTTATGACATCAAGGAAGAAAGTTC	Plasmid construction
Rab7-R	CGGGGTACCGTGCAGCTACAAGTCTCTGC	Plasmid construction
TRIF-F	CCCAAGCTTCGCCACCATGGCAGATGGTGGAGTAGAG	Plasmid construction
TRIF-R	CGCGGATCCCGAGAATCAAACCCATTGGGCGAG	Plasmid construction
MyD88-F	CCCAAGCTTCGCCACCATGGCATCAAAATCAAGTATAGAC	Plasmid construction
MyD88-R	CGCGGATCCCGTTGAAAAGATCGGGGCAGTGC	Plasmid construction
TIRAP-F	CCCAAGCTTCGCCACCATGGAGGAAGACGCGTCAG	Plasmid construction
TIRAP-R	CGCGGATCCCGGCCGTCAGATTGAGATGCAC	Plasmid construction
EPC-*ifn-1*-F	ATGAAAACTCAAATGTGGACGTA	Real-time PCR
EPC-*ifn-1*-R	GATAGTTTCCACCCATTTCCTTAA	Real-time PCR
EPC-*Viperin*-F	AGCGAGGCTTACGACTTCTG	Real-time PCR
EPC-*Viperin*-R	GCACCAACTCTCCCAGAAAA	Real-time PCR
EPC-*β-actin*-F	GCCGTGACCTGACTGACTACCT	Real-time PCR
EPC-*β-actin*-R	GCCACATAGCAGAGCTTCTCCTTG	Real-time PCR

### Bioinformatics analysis of *Cc*Tlr19

Multiple sequence alignment to identify the functional domain of the *Cc*Tlr19 protein was performed with Clustal W. The SWISS-MODEL database was used to predict the structures of TLR. The phylogenetic tree was established by MEGA 6.0 software using the neighbor-joining method. The GenBank accession numbers are shown in Additional file 1.

### Isolation and stimulation of common carp head kidney leukocytes (HKL)

Head kidney tissue was aseptically excised from common carp to gently push through a 100-μm nylon mesh and density gradient centrifugation with 34% and 51% Percoll (Sigma-Aldrich, Germany) as described previously [31]. Approximately 10^7^ cells/well were seeded in 24-well plates with 500 μL of L-15 complete medium (Gibco). After recovering overnight at 25 °C, drug treatment was performed using LPS (10 μg/mL), peptidoglycan (PGN) (10 μg/mL), flagellin (10 ng/mL) and poly(I:C) (5 ng/mL). Samples were collected at different time periods. *Cctlr19* mRNA expression was detected by qPCR.

### RNA extraction, reverse transcription and quantitative real-time PCR

Total RNA from primary cells, EPC cells or tissues was extracted using RNA simple Total RNA kit (Tiangen Biotech, China) according to the manufacturer’s instructions. Reverse transcription of RNA and synthesis of first-strand cDNA were performed using a Fast Quant Kit (with gDNase) (Tiangen) following the manufacturer’s protocol. qPCR was used to detect gene expression and performed on a LightCycler 96 instrument (Roche, Switzerland) using TransStart Tip Green qPCR Supermix (TransGen Biotech, China). The qPCR procedure was as follows: 94 °C for 30 s followed by 40 cycles of 94 °C for 5 s and 60 °C for 30 s. For gene expression in tissue and primary cells, 40S ribosomal protein S11 was used as an internal reference. For EPC cells, gene expression was corrected by β-actin. The primers used are shown in Table 1.

### Confocal fluorescence microscopy

HeLa or EPC cells were seeded onto coverslips in a 24-well plate. The following day, the cells were transfected with target plasmids using transfection reagent. After 48 h, the cells were washed twice with PBS, fixed with 4% paraformaldehyde (PFA) for 30 min and then blocked with PBS containing 1% BSA. For the subcellular localization of Tlr19 in the resting state, the cells were incubated with mouse anti-FLAG (Sigma-Aldrich, 1:800) or endoplasmic reticulum (ER)-marker calnexin (1:1000, Abcam, UK). After that, the cells were treated with the indicated fluorescent coupled secondary antibody. Then, nucleus was stained with DAPI. Finally, the stained cells were viewed under a laser confocal scanning microscope and analyzed with ImageJ software.

### Luciferase activity assays

293 T cells in 96-well plates were co-transfected with expression plasmids as required: rhRL-TK and Luci-*Ccifns*. For each transfection, the total amount of DNA was balanced by the addition of an empty vector. After transfection for 48 h, the cells were lysed with Dual-Glo^®^ luciferase reagent (Promega). The supernatant was used to measure the activity of *Firefly* and *Renilla* luciferase according to the instructions of the manufacturer. All the experiments were performed in triplicate.

### Western blotting and PNGase F digestion

Epithelioma papulosum cyprinid cells were transfected with empty vector or a Tlr19-carrying plasmid. After 24 h, the cells were lysed with 1  ×  SDS-PAGE loading buffer. The whole-cell lysate was divided into two groups, one with and one without PNGase F (New England Biolabs, USA), according to the manufacturer’s instructions. Briefly, the sample was mixed with PNGase F at 37 °C for 1 h. The proteins in the PNGase F-digested group and the control group were isolated by 10% SDS-PAGE and transferred to nitrocellulose membranes. The membranes were blocked with 5% nonfat milk. The proteins were probed with different antibodies. The primary antibody, the anti-GFP monoclonal antibody (Solarbio, China), was diluted at 1:1000, and HRP-conjugated anti-rabbit IgG (Proteintech, USA) was diluted at 1:5000. The immunoreactive proteins were detected using a chemical luminescence substrate with an Amersham Imager 600. The results are representative of data from three independent experiments.

### Statistical analysis

Statistical analysis was carried out using GraphPad Prism 7.0 software (GraphPad, La Jolla, CA, USA). The results of three independent experiments are expressed as the means  ±  SD. Data were processed using one- or two-way ANOVA or Tukey test. *P* values of less than 0.05 were considered statistically significant (*P  <  0.05, **P  <  0.01, ***P  <  0.001, ****P  <  0.0001).

## Results

### Cloning and sequence analysis of *Cctlr19*

**Figure 1:**
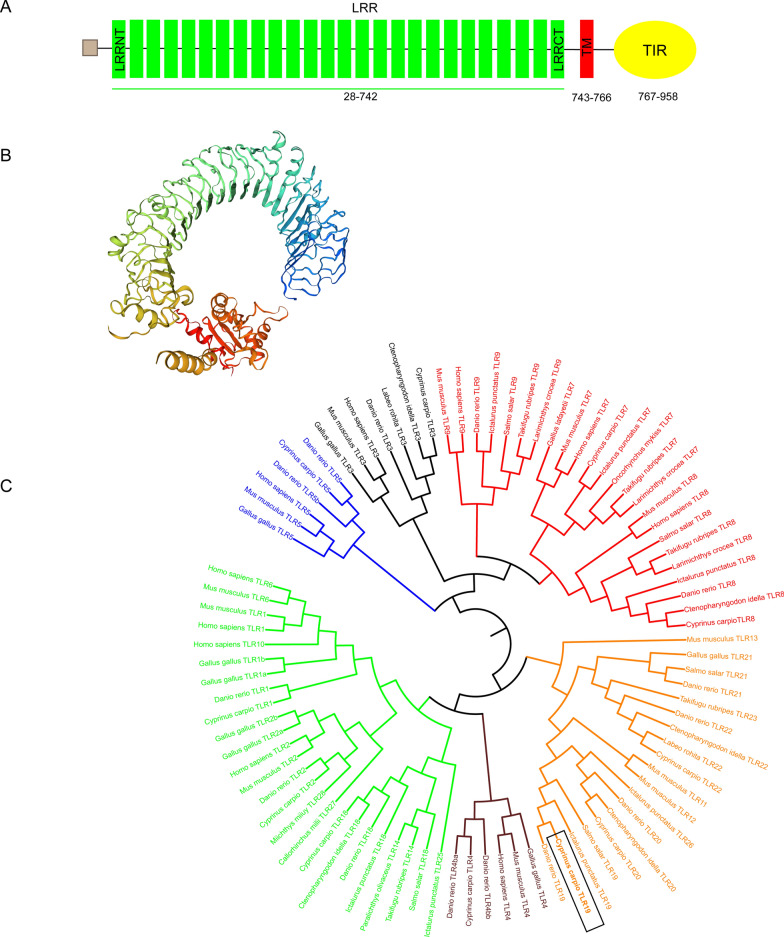
**Cloning of the full-length cDNA of *Cctlr19*.** **A** A schematic diagram showing the domain architecture of common carp Tlr19. The gray box represents the signal peptide. The leucine-rich-repeat (LRR) regions are shown with green rectangles. The transmembrane (TM) domains are shown with red rectangles, and Toll/interleukin-1 receptor (TIR) domains are shown as yellow ovals. **B** Modeled three-dimensional structure of common carp Tlr19 shown as a cartoon. **C** Phylogenetic analysis of Tlr19 amino acid sequences. The phylogenetic tree was constructed using multiple alignment of amino acids generated by the neighbor-joining method in the MEGA 6.0. program. Green, black, purple, blue, red and orange color represents the TLR1, 3, 4, 5, 7 and 11 subfamilies, respectively, while the black box indicated the *Cc*Tlr19. The GenBank accession numbers of TLR sequences are shown in Additional file 1.

In the present study, we cloned and identified a novel *tlr19* cDNA sequence from common carp named *Cctlr19*. Full-length *Cctlr19* cDNA (GenBank accession No. MW411431) was 3160 bp, including a 5’′UTR of 26 bp and a 3’UTR of 260 bp. The largest open reading frame was 2874 bp and encoded a peptide of 957 amino acids with a molecular weight of 109 557 Da. SWISS-MODEL prediction and sequence alignment show that the *Cc*Tlr19 protein exhibited typical TLR domains, including a signal peptide, a 26 leucine-rich repeat domain, a transmembrane region and a Toll-interleukin-1 receptor (TIR) domain (Figures 1A, B; Additional file 2). Phylogenetic analysis revealed that *Cc*Tlr19 clustered with other Tlr19 from *Danio rerio*, *Ictalurus punctatus* and *Salmo salar* and showed the closest relationship with zebrafish Tlr19 (73.4%) (Figure 1C).

### Tissue expression profile and subcellular localization of *Cc*Tlr19

**Figure 2:**
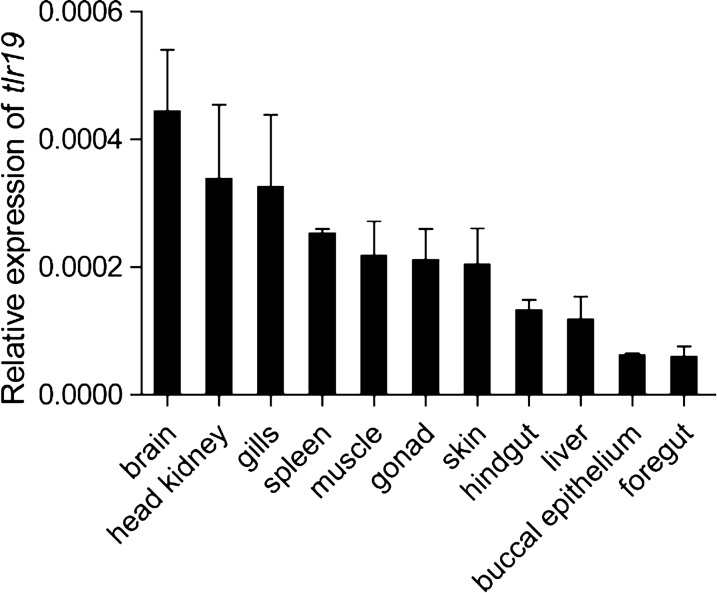
**Tissue expression of *Cctlr19* in normal common carp.** The expression of *Cctlr19* mRNA in the liver, spleen, head kidney, foregut, hindgut, skin, gills, gonad, muscle, buccal epithelium and brain was detected by qPCR. 40S ribosomal protein S11 in each tissue was amplified as an internal control, *n * =  3.

To investigate the expression profile of *Cctlr19* in healthy tissue, qPCR analysis was performed. Transcripts of *Cctlr19* were ubiquitously detected in all the examined tissues, with the highest levels in the brain and head kidney and the lowest level in the foregut (Figure 2).

**Figure 3:**
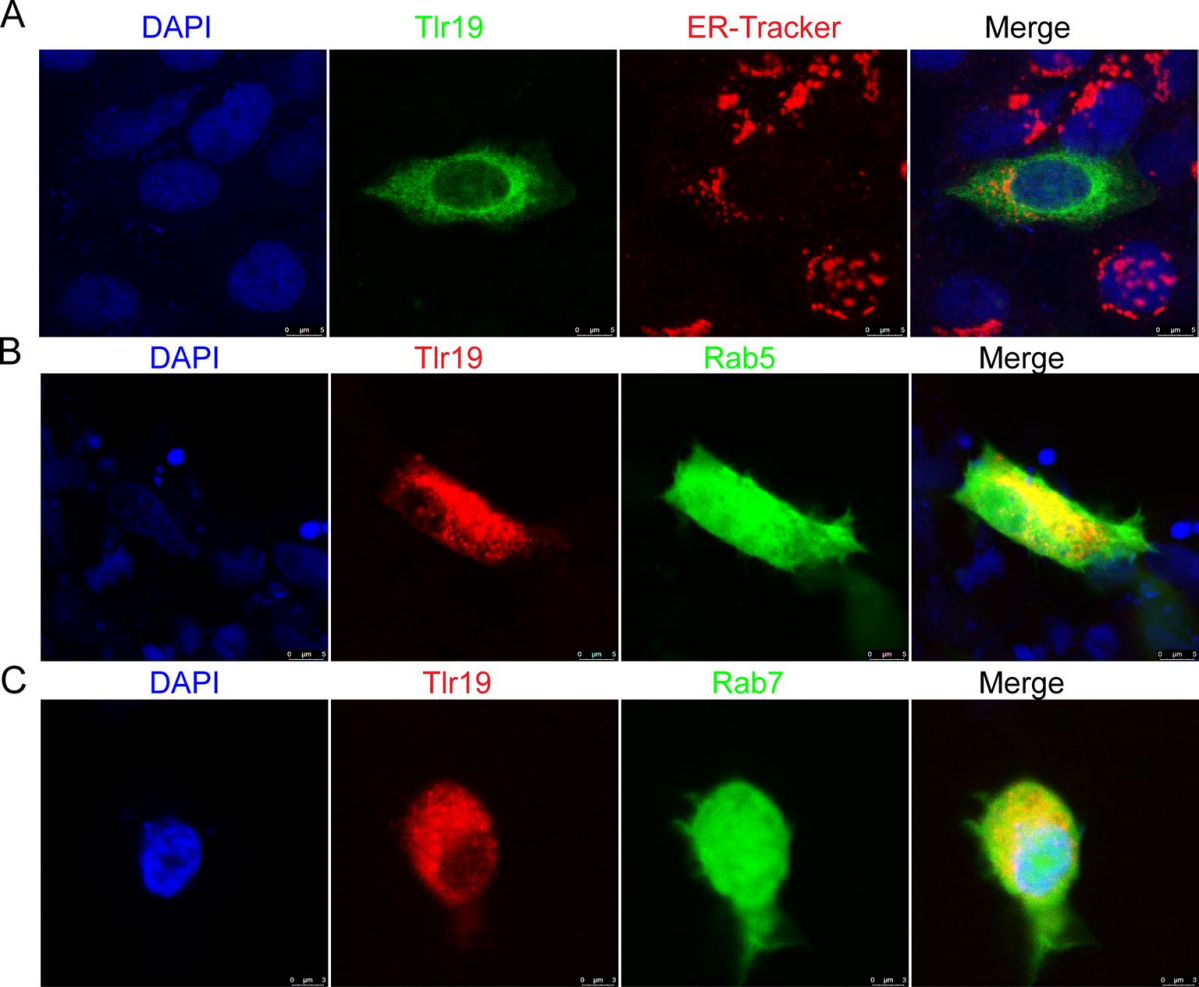
**Localization of *Cc*Tlr19.** **A** For *Cc*Tlr19 colocalization with the endoplasmic reticulum, EPC cells were transiently transfected with Tlr19-GFP. After 24 h, the cells were fixed with 4% paraformaldehyde, stained with ER-Tracker, and then treated with Cy3-conjugated (red) goat anti-rabbit IgG secondary antibody (Ab). The red signal represents the ER, the green signal indicates *Cc*Tlr19, and blue staining indicates the nucleus. For *Cc*Tlr19 colocalization with endosomes, EPC cells transiently co-transfected with Tlr19-FUGW, Rab5-GFP (early endosome marker) **B** or Rab7-GFP (late endosome marker) **C**, and the cells were stained with mouse anti-FLAG antibody (Ab). The cells were treated with Cy3-conjugated secondary Ab and DAPI and then measured by immunofluorescence confocal microscopy. Red signals represent overexpressed *Cc*Tlr19, green signals display overexpressed endosomes, and blue staining indicates the nucleus.

To gain a better understanding of *Cc*Tlr19 functions, the subcellular localization was investigated. We transfected EPC cells with GFP-tagged *Cc*Tlr19 and then stained with ER-Tracker (calnexin). As illustrated in Figure 3, Tlr19 largely merged with Rab5 (an early endosome marker) and did not colocalize with the endoplasmic reticulum, implying that Tlr19 is synthesized in ribosomes and does not bind to the ER. Then, Tlr19 moves on to early endosomes.

### Expression modulation of *Cctlr19* following *A. hydrophila* and poly(I:C) stimulation

**Figure 4:**
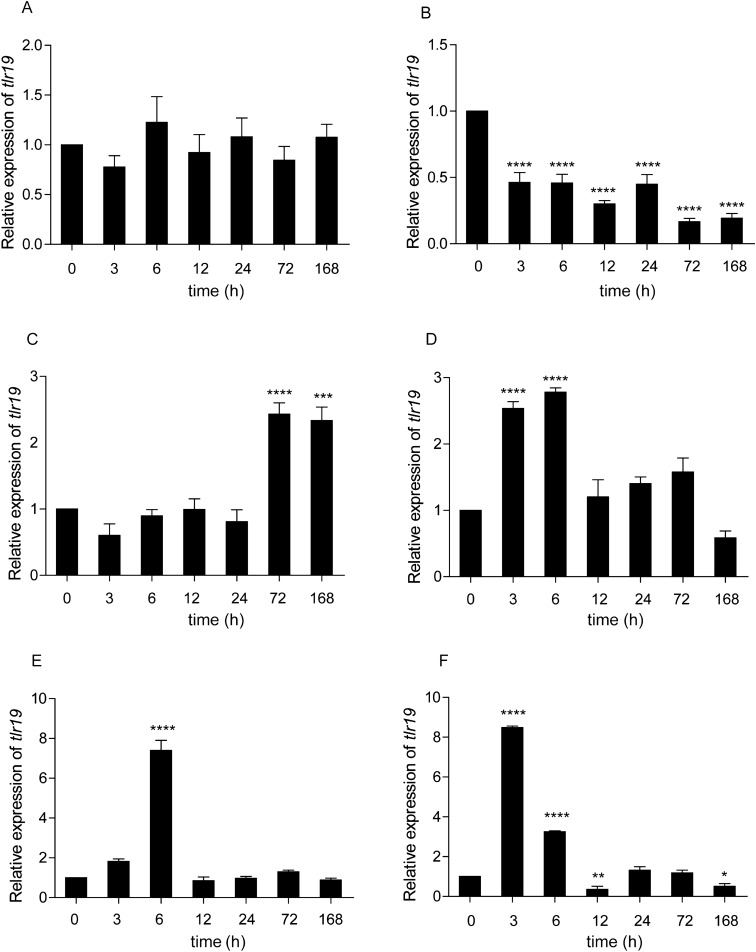
**The relative expression of *Cctlr19* in various tissues of common carp after i.p. injection with *A. hydrophila*.** The expression of *Cctlr19* in the liver (**A**), spleen (**B**), head kidney (**C**), foregut (**D**), hindgut (**E**) and skin (**F**) at different time points is shown. The results were calculated relative to the expression of the 40S ribosomal protein S11 gene. Data are presented as a fold increase compared to the unstimulated control group (denoted by 0 h). Means  ±  SD (*n * =  3), *P  <  0.05, **P  <  0.01, ***P  <  0.001, ****P  <  0.0001, one-way ANOVA.

To understand the modulation of *Cctlr19* expression, qPCR analysis was performed in six different tissues (i.e., liver, spleen, head kidney, foregut, hindgut, and skin) after intraperitoneal injection with inactivated *A. hydrophila* and poly(I:C). As illustrated in Figure 4, significant upregulation of *Cctlr19* was observed in the head kidney, foregut, hindgut and skin upon stimulation. The expression level of *Cctlr19* in the head kidney was induced and peaked (2.4-fold) at 72 h (Figure 4C). In the foregut and hindgut, *Cctlr19* expression was induced at 3 h and reached its highest value at 6 h (2.8-fold and 7.4-fold, respectively) (Figures 4D, E). In the skin, *Cctlr19* expression was induced and peaked at 3 h (8.5-fold) (Figure 4F). In contrast, no marked change in *Cctlr19* expression was observed in the liver at any time points post challenge (Figure 4A). However, in the spleen, the expression of *Cctlr19* was downregulated (Figure 4B).

**Figure 5:**
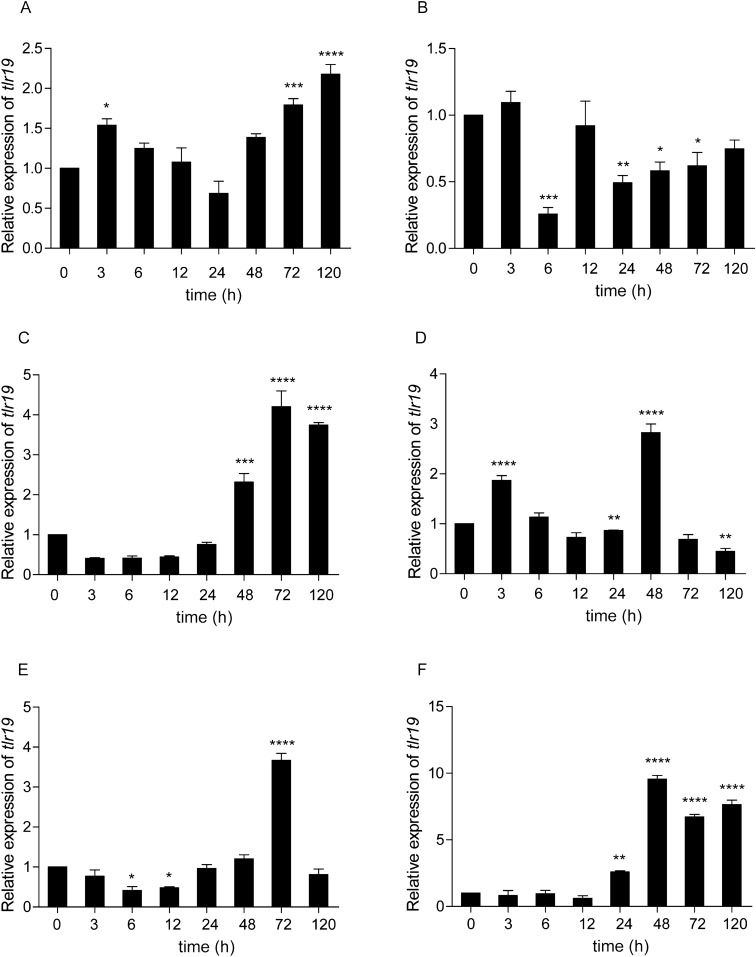
**The relative expression of *Cctlr19* in various tissues of common carp after i.p. injection with poly(I:C).** The expression of *Cctlr19* in the liver (**A**), spleen (**B**), head kidney (**C**), foregut (**D**), hindgut (**E**) and skin (**F**) at different time points is shown. The results were calculated relative to the expression of the 40S ribosomal protein S11 gene. Data are presented as a fold increase compared to the unstimulated control group (denoted by 0 h). Means  ±  SD, (*n * =  3), *P  <  0.05, **P  <  0.01, ***P  <  0.001, ****P  <  0.0001, one-way ANOVA.

To investigate the role of *Cc*Tlr19 in host defense against viruses, a double-stranded RNA mimic, poly(I:C), was used to stimulate common carp, and the mRNA expression levels of *Cctlr19* were measured. As shown in Figure 5, the expression of *Cctlr19* was significantly upregulated in the liver, head kidney, foregut, hindgut, and skin. In the liver, the expression of *Cctlr19* was induced at 3 h and peaked at 120 h (2.2-fold) (Figure 5A). In the head kidney, *Cctlr19* mRNA expression increased at 48 h, reaching the highest level at 72 h (4.2-fold) (Figure 5C). In the foregut, *Cctlr19* mRNA first showed a small peak at 3 h, then began to decrease at 6 h, and reached a peak value at 48 h (2.8-fold) (Figure 5D). In the hindgut, the expression of *Cctlr19* was downregulated at 6 h, then increased a peak value at 72 h (3.7-fold) (Figure 5E). The expression level of *Cctlr19* in the skin was induced at 24 h and peaked at 48 h (9.6-fold) (Figure 5F). In the spleen, *Cctlr19* gene expression showed no significant increase after poly(I:C) stimulation but showed a decreasing trend (Figure 5B).

### Induced expression of *Cctlr19* in HKL

**Figure 6:**
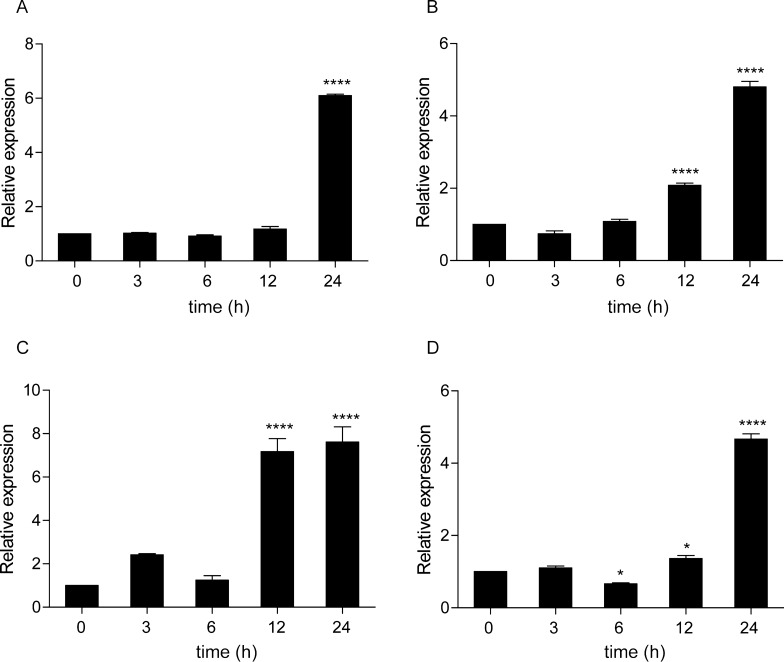
**The relative expression of *Cctlr19* in the HKL of common carp after treatment with poly(I:C).** (**A**), LPS (**B**), PGN (**C**) and flagellin (**D**) at different time points. The results were calculated relative to the expression of the 40S ribosomal protein S11 gene. The data are presented as a fold increase compared to the unstimulated control group (denoted by 0 h). Means  ±  SD (*n * =  3), *P  <  0.05, **P  <  0.01, ****P  <  0.0001, one-way ANOVA.

Then, we isolated leukocytes from the head kidney of common carp. The expression level of *Cctlr19* was upregulated after stimulation with poly(I:C), LPS, PGN and flagellin. As shown in Figure 6A, *Cctlr19* expression was induced and reached a peak level (7.0-fold) at 24 h after poly(I:C) stimulation. When challenged with LPS and flagellin, the expression of *Cctlr19* was induced at 12 h and peaked at 24 h (4.7-fold and 4.8-fold, respectively) (Figure 6B, D). *Cctlr19* expression was induced and reached a peak value at 24 h (7.6-fold) with PGN stimulation (Figure 6C).

These preliminary results indicate that *Cc*Tlr19 might be involved in antibacterial and antiviral immune responses.

### Tlr19 recruits TRIF as an adaptor

**Figure 7:**
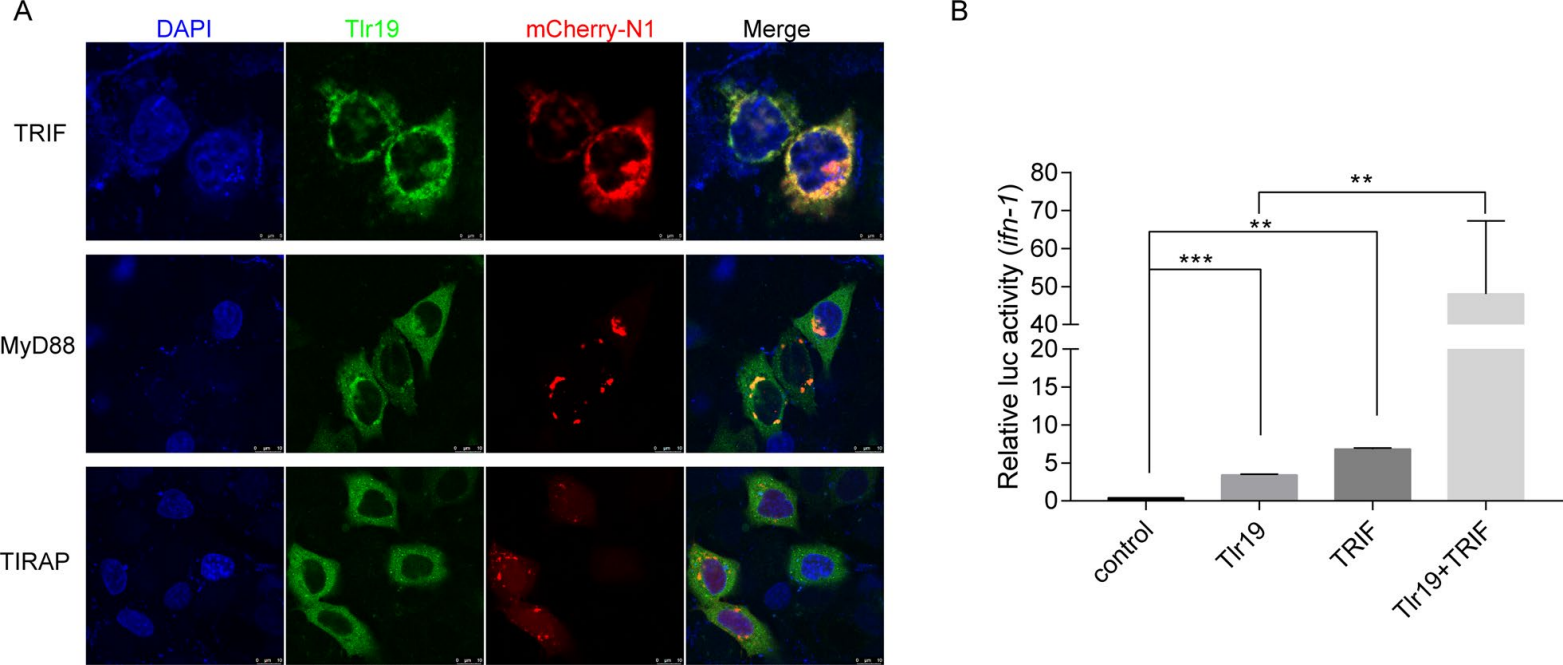
**Tlr19 colocalizes and interacts with TRIF.** **A** HeLa cells transiently transfected with Tlr19-GFP and TRIF-mCherry-N1, MyD88-mCherry-N1 or TIRAP-mCherry-N1. After 24 h, the cells were fixed with 4% paraformaldehyde, stained with DAPI and subjected to confocal microscopy analysis. Green signals represent *Cc*Tlr19, red signals display adaptors, and blue staining indicates the nucleus. **B** 293 T cells were transfected with empty vector, Tlr19, TRIF or Tlr19  +  TRIF together with Luci-*ifn* and rhRL-TK reporter plasmids. After 48 h of transfection, all luciferase activities were calculated by normalization to *Renilla* luciferase activity, and the results are shown as the fold change compared to the control group (empty vector). Means  ±  SD (*n * =  3), **P  <  0.01, ***P  <  0.001, one-way ANOVA.

Previous studies have shown that once TLR are activated, the TIR domain recruit adaptors, and downstream signaling is initiated. To further explore the adaptor recruited by *Cc*Tlr19, we co-transfected cells with GFP-tagged *Cc*Tlr19 and mCherry-tagged adaptors (TRIF, MyD88 and TIRAP). The fluorescence clearly shows that Tlr19 is colocalized with TRIF but not with other adaptors (Figure 7A). For further exploration, *Cc*Tlr19 and TRIF were co-transfected into 293 T cells together with the *ifn-1* reporter plasmid. The results indicate that overexpression of *Cc*Tlr19 or TRIF potently increased *ifn-1* activity. Furthermore, *ifn-1* activity was enhanced in 293 T cells co-transfected with *Cc*Tlr19 and TRIF (Figure 7B), demonstrating that *Cc*Tlr19 activated *ifn* activity by recruiting TRIF.

### *Cc*Tlr19 promotes the expression of *ifns*

**Figure 8:**
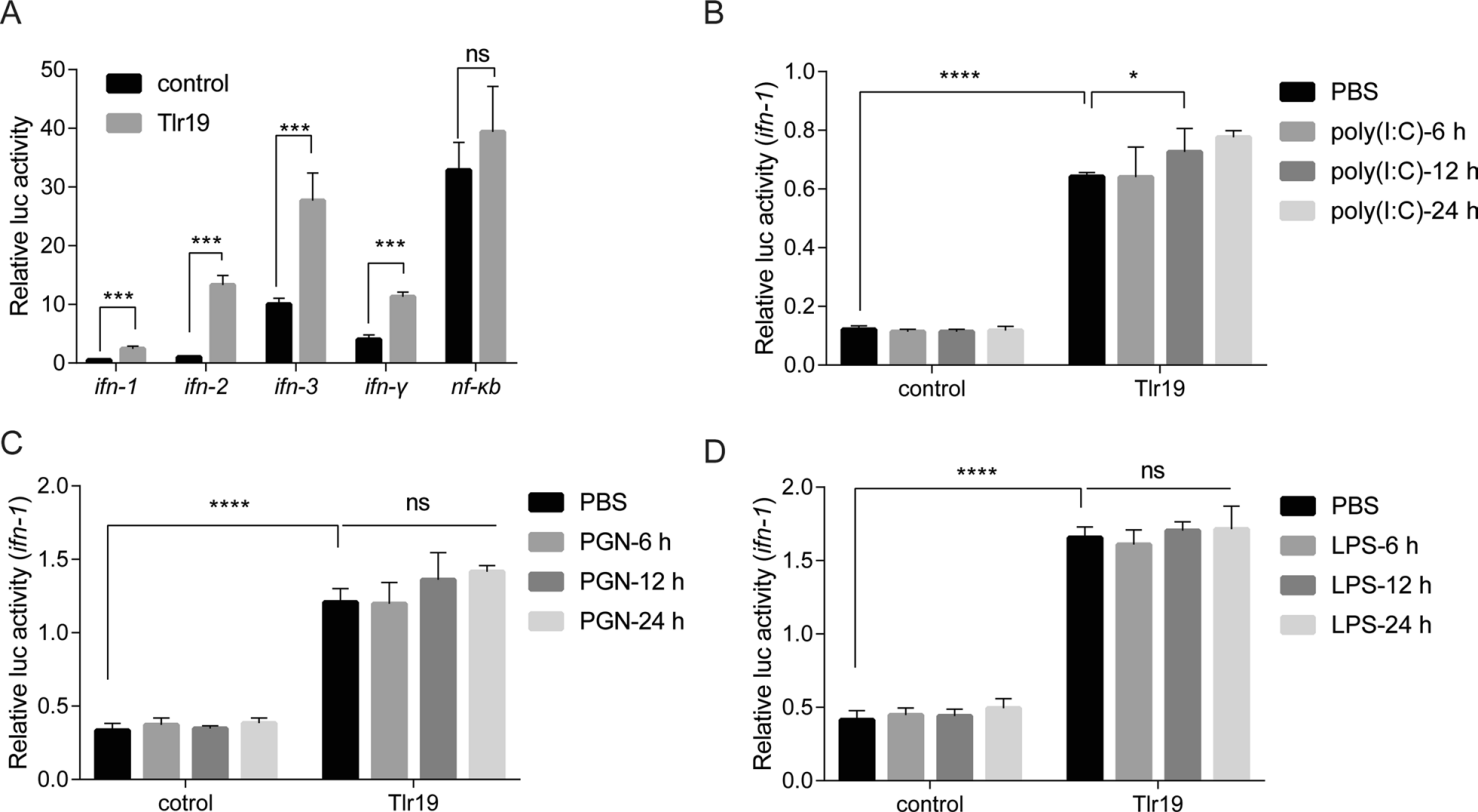
**Activation of *ifn* mediated by *Cc*Tlr19.** **A** Luci-*ifn* and *nf-kb* reporter plasmids, rhRL-TK and *Cc*Tlr19-FUGW expression vector or an empty vector (control) were transfected into 293 T cells. After 48 h, *Firefly* and *Renilla* luciferase activities were detected, and the ratio was calculated. Means  ±  SD (*n * =  4), *P  <  0.05, ***P  <  0.001, ****P  <  0.0001, *t* test. **B**–**D** 293 T cells were transfected targeted plasmid. After 36 h, the cells were stimulated with poly(I:C), PGN and LPS for the indicated periods of time. *Firefly* and *Renilla* luciferase activities were detected, and the ratio was calculated. Means  ±  SD (*n * =  4), *P  <  0.05, ***P  <  0.001, ****P  <  0.0001, two-way ANOVA.

IFN and NF-κB are recognized as important molecules involved in TLR-mediated signaling. Then, luciferase reporter assays were performed to examine the promoter activities of IFN and NF-κB upon Tlr19 overexpression. As shown in Figure 8A, except for *nf-κb*, the luciferase activities of all the examined *ifns* (*ifn-1*, *ifn-2*, *ifn-3* and *ifn-γ*) were significantly increased in Tlr19-overexpressing cells. Furthermore, the luciferase activity of *ifn-1* was more pronounced in the case of poly(I:C)-infected cells at 12 h, while LPS and PGN did not activate *ifn-1* (Figures 8B–D).

### Effect of *Cc*Tlr19 on cytokine expression in EPC cells

**Figure 9:**
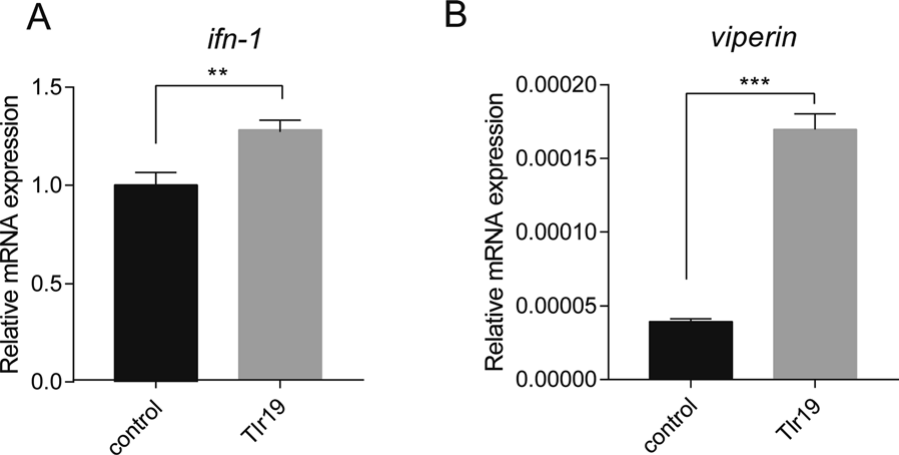
***Cc*Tlr19 induces the expression of cytokines. ***Cc*Tlr19 was overexpressed in EPC cells in 24-well plates, and RNA was extracted using TRIzol. qPCR was used to test the expression of *ifn-1* and *viperin*. The results were calculated relative to the expression of β-actin using qPCR. Mean  ±  SD (*n * =  3), **P  <  0.01, ***P  <  0.001, *t* test.

To investigate the involvement of *Cc*Tlr19 in inducing cytokines, we analyzed the gene expression levels of *ifn-1* and *viperin*. As shown in Figure 9, the expression of *ifn-1* (Figure 9A) and *viperin* (Figure 9B) was significantly increased in EPC cells compared with that in the control group.

### *Cc*Tlr19 is modified by N-linked glycosylation

**Figure 10:**
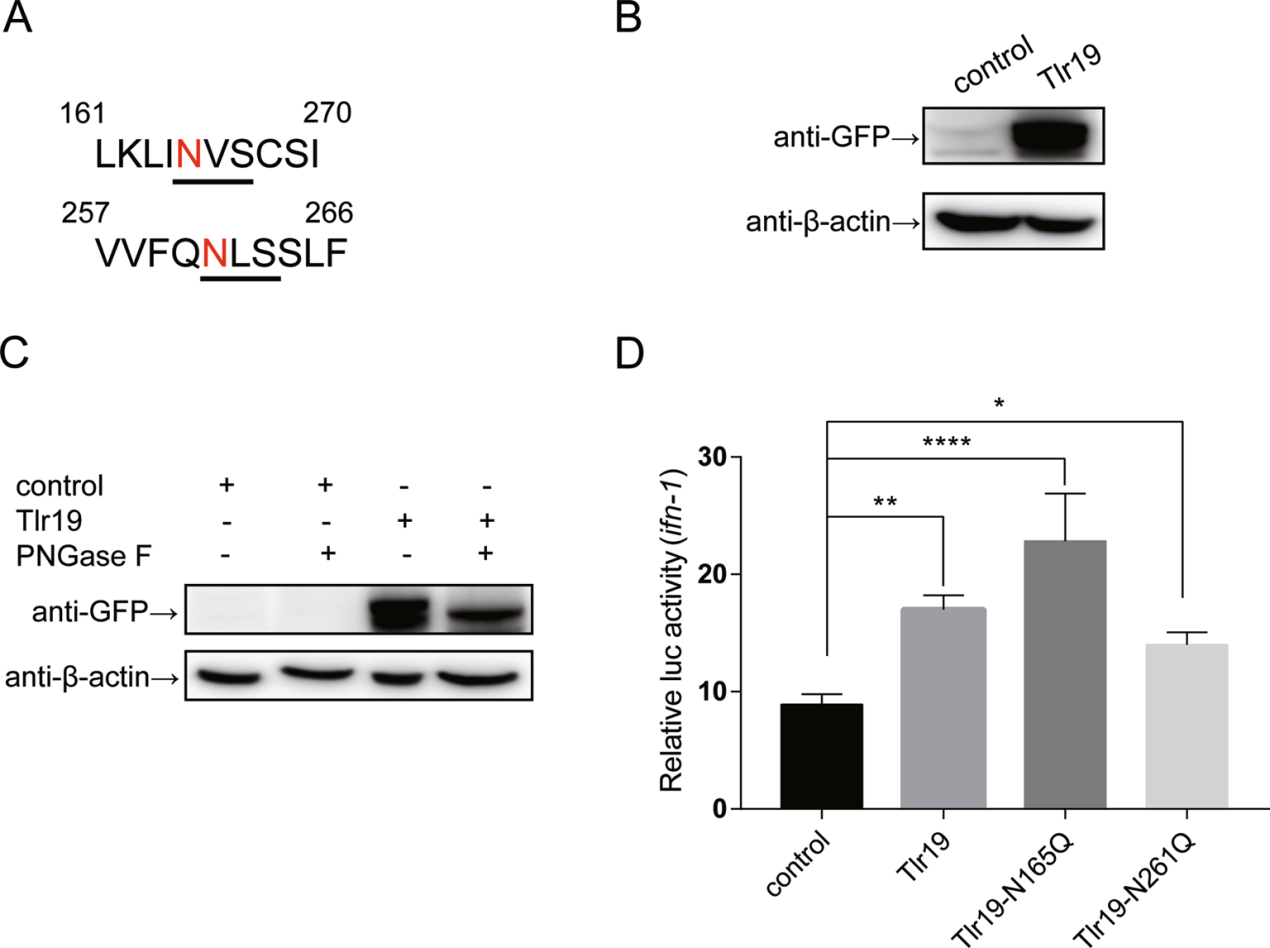
***Cc*Tlr19 is modified with N-linked glycosylation.** **A** N-X-S/T consensus sequence of *Cc*Tlr19. **B** EPC cells seeded in 6-well plates were transfected with the indicated plasmids (*Cc*Tlr19-GFP or an empty vector) separately. After 48 h, the whole-cell lysate was subjected to immunoblot assay with anti-GFP and anti-β-actin Abs. **C** Glycosidase digested *Cc*Tlr19. EPC cells seeded in 6-well plates were transfected with *Cc*Tlr19 or an empty vector, and the whole-cell lysate was digested with PNGase F or not (control). **D** A Luci-*ifn-1* plasmid, rhRL-TK, together with *Cc*Tlr19 expression vector or an empty vector (control) were transfected into 293 T cells. After 48 h, *Firefly* and *Renilla* luciferase activity was detected, and the ratio was calculated. Means  ±  SD (*n * =  4), *P  <  0.05, **P  <  0.01, ***P  <  0.001, ****P  <  0.0001, one-way ANOVA.

Furthermore, the conserved motif (N-X-S/T) was observed in the asparagine residues 165 and 261 of *Cc*Tlr19 (Figure 10A), implying that Tlr19 may undergo glycosylation modification. As illustrated in Figure 10B, two bands appeared in the blots of *Cc*Tlr19-overexpressing EPC cells, and it was speculated that the larger band of *Cc*Tlr19 might be its glycosylated form. To test this hypothesis, the whole-cell lysate of *Cc*Tlr19-overexpressing EPC cells was digested with PNGase F, after which only one band was apparent (Figure 10C). In addition, we constructed two mutants of *Cc*Tlr19 (*Cc*Tlr19-N165Q and *Cc*Tlr19-N261Q) and tested the luciferase activity of *ifns* in cells carrying one of the mutants. As shown in Figure 10D, *Cc*Tlr19-N165Q and *Cc*Tlr19-N261Q induced the luciferase activity of *ifn-1*. These results demonstrate that *Cc*Tlr19 undergoes N-linked glycosylation and that glycosylation is not crucial for antiviral property.

## Discussion

In the mid-1990s, the discovery of Toll-like receptors (TLR) showed that pathogen recognition in the innate immune system was specific, relying on pattern-recognition receptors (PRR) [32]. Tlr19 is considered to be a fish-specific TLR [33] and plays a vital role in bacterial and viral recognition.

In the present study, we analyzed the structure and evolutionary relationship of Tlr19 in the common carp. *Cc*Tlr19 appears homologous to known fish Tlr19. Structural analysis revealed that *Cc*Tlr19 has a typical TLR structure (Figure 1A), including a signal peptide, a 26-LRR motif, a transmembrane region and a TIR domain. The extracellular LRR domain is important for direct binding [34] and is generally highly conserved in each TLR subfamily [19]. The number of LRR motifs in *Cc*Tlr19 is close to that of zebrafish (24-LRR motif) [33] and Atlantic salmon (26-LRR motif) [15]. Although common carp Tlr3 and Tlr22 have similar amounts of the LRR motif, the 3D structure shows that *Cc*Tlr19 was different from Tlr3 and Tlr22 (Additional file 3). Multiple sequence alignments show that the TIR domains of *Cc*Tlr19 shared high identity with other fish. In addition, phylogenetic analysis revealed that *Cc*Tlr19 belonged to the Tlr11 subfamily, clustered with other fish Tlr19 and was highly similar to zebrafish Tlr19 (Figure 1C). These findings suggest that *Cc*Tlr19 might exert similar functions as Tlr19 in other fishes.

*Cctlr19* was found to be widespread among tissues, which is similar to its distribution in Atlantic salmon and yellow catfish [15, 18]. Surprisingly, the highest level of *Cctlr19* expression was not in the spleen, which is different than that in other teleosts. For example, in Atlantic salmon, Tibet fish and yellow catfish, *tlr19* is expressed at the highest level in the spleen [15, 16, 18]. In the present study, *Cctlr19* was expressed in a wide range of tissues but at relatively high levels in spleen tissue. However, a high expression level of *Cctlr19* was observed in the brain tissue, which was similar to that of common carp *tlr1* [22] and gibel carp *tlr2* [35]. In addition, high expression of *Cctlr19* was also detected in the head kidney and gills, which was similar to that of Atlantic salmon [15]. The different expression patterns of *tlr19* in healthy fish indicate that the regulation of *tlr19* may be the result of species variations, individual status, developmental stage and genetic background [36]. In addition, the subcellular location of TLR is relevant to ligand identification, and intracellular TLR are restricted to recognized nucleic acid ligands [37]. Our results show that *Cc*Tlr19 is localized in the intracellular compartment (Figure 3), which is consistent with salmon and grass carp Tlr19 [15, 20]. As a consequence, *Cc*Tlr19 is an intracellular Tlr.

Toll-like receptor 19 was reported to be involved in innate immunity when infected with microbial pathogens. For instance, the expression of yellow catfish *tlr19* was upregulated in immune-related tissues after challenge with *A. hydrophila* [18]. In channel catfish, the expression of *tlr19* was significantly upregulated in the liver and spleen by exposure to *Edwardsiella ictalurid* [38]. In the present study, the expression of *Cctlr19* was induced by *A. hydrophila.* The results demonstrate that *tlr19* was involved in fish immunity against bacteria, although different antibacterial patterns may be involved in different tissues and in various fish. Poly(I:C) was used as a model of an infective double-stranded genome virus. The expression of *Cctlr19* was upregulated in the liver, head kidney, foregut, hindgut and skin (Figure 5). Similarly, the mRNA level of grass carp *tlr19* was significantly upregulated 48 h post-GCRV infection [20]. Fish live in water, which may contain RNA viruses and RNA products of microbial origin. During evolution, vertebrates in water may have developed numerous RNA-sensing TLR and IFN systems to protect against these pathogens, as these systems are different than those in land animals [12, 39]. In addition to fish *tlr19*, other fish *tlrs*, including *tlr3* and *tlr22*, can be regulated by poly(I:C), a mimic of viral dsRNA [12, 40, 41]. Furthermore, leukocytes consist of heterogeneous cells [42] and are widely used as experimental systems to study immune responses [43]. After challenge with the viral mimic poly(I:C), the expression level of *tlr19* was significantly upregulated in isolated peripheral blood lymphocytes of yellow catfish [18]. In the current study, *Cctlr19* expression increased after stimulation with different ligands in head kidney leukocytes (HKL) (Figure 6), which further confirmed the in vivo results. These results reveal that *Cc*Tlr19 participate in antibacterial and antiviral innate immunity.

Once TLR recognize PAMP, the intracellular TIR domain recruits adaptors [44]. To date, seven adaptors of TLR have been identified in mammals [45]. Previous studies have reported that intracellular TLR can interact with TRIF as adaptors. For example, mammalian TLR3, TLR7 and grass carp Tlr19 recruits the molecule TRIF as the adaptor [20, 46, 47]. In this study, *Cc*Tlr19 recruited TRIF as an adaptor (Figure 7), similar to other intracellular Tlr.

The TRIF-dependent pathway exists in both mammals and fish, triggering the expression of *ifn* and interferon-stimulated genes [48, 49]. Grass carp Tlr19 facilitates the expression of *ifn* by recruiting TRIF [20]. Similarly, *Cc*Tlr19 recruits TRIF and activates the luciferase of *ifn* (Figs. 7,  8). *Viperin* and MX2 are IFN-inducible proteins that can interfere with the replication of diverse viruses [50, 51]. TLR19 overexpression significantly induced the expression of *mx2* in grass carp [20]. In this study, the expression of *ifn-1* and *viperin* was upregulated in *Cc*Tlr19-overexpressing EPC cells (Figure 9). Collectively, *Cc*Tlr19 recruits TRIF to trigger *ifn-1* expression, which is required for the innate immune response.

Among posttranslational modifications, glycosylation is a major modification of eukaryotic cells that help proteins fold correctly [52]. N-linked glycosylation is the most common glycosylation type, in which the glycan chain has a conserved motif of N-X-S/T [53]. Multiple alignment analysis show that *Cc*Tlr19 had two conserved glycosylation sites (Figure 10A). Glycosidase digestion verified that *Cc*Tlr19 was modified by N-linked glycosylation (Figure 10B). Previous studies showed that changes in one of the asparagine residues did not affect TLR3-dependent activation of the reporter assay; however, mutations in 2 of the 15 glycosylation sites (N247 and N413) gave rise to a nonfunctional TLR3 [54]. In the current study, wild-type and un-glycosylated mutants (N165Q or N261Q) of *Cc*Tlr19 separately increased the luciferase activity of *ifn-1*, which was similar to that of TLR3. However, the function of the two mutations in the glycosylation sites of *Cc*Tlr19 remains to be further studied.

In conclusion, *Cc*Tlr19 is a typical member of the fish-specific TLR family. *Cc*Tlr19 participates in antibacterial and antiviral immunity. Moreover, *Cc*Tlr19 recruits the adaptor TRIF and induces the expression of *ifn-1* and *viperin*. This study provides a better understanding of the mechanism of Tlr19 in fish innate immunity.

## Supplementary Information

**Additional file 1.** The TLR protein sequences used in this study.

**Additional file 2.** The multiple alignment analysis of *Cc*Tlr19. The sequences were aligned using the Clustal W method. Identical, conserved and similar substituted amino acid residues are indicated in (*), (: or .), respectively.

**Additional file 3.** Modeled three-dimensional structure of *Cc*TLR19, *Cc*Tlr3 and *Cc*Tlr22 ectodomain in a cartoon mode.

## Data Availability

The dataset supporting the conclusions of this article is available in GenBank with accession number MW411431.
