# Supplementary material for: Identification and functional characterization of a fish-specific tlr19 in common carp (Cyprinus carpio L.) that recruits TRIF as an adaptor and induces ifn expression during the immune response
Source: Vet Res. 2021 Jun 15;52:88. doi: 10.1186/s13567-021-00957-3 (PMC8207781; doi:10.1186/s13567-021-00957-3)
Supplement: Supplementary file 1 — Additional file 1. The TLR protein sequences used in this study. [file 13567_2021_957_MOESM1_ESM.docx]

**Additional file 1. The TLR protein sequences used in this study.**

| **Species** | **Protein** | **Accession number** |
| --- | --- | --- |
| *Danio rerio*  *Ictalurus punctatus*  *Salmo salar*  *Ictalurus punctatus*  *Danio rerio*  *Cyprinus carpio*  *Ctenopharyngodon Idella*  *Mus musculus*  *Mus musculus*  *Danio rerio*  *Ctenopharyngodon idella*  *Cyprinus carpio*  *Takifugu rubripes*  *Mus musculus*  *Gallus gallus*  *Danio rerio*  *Salmo salar*  *Homo sapiens* | TLR19  TLR19  TLR19  TLR26  TLR20  TLR20  TLR20  TLR11  TLR12  TLR22  TLR22  TLR22  TLR23  TLR13  TLR21  TLR21  TLR21  TLR10 | XP_002664892.4  AEI59675.1  CDH93609.2  AEI59681.1  NP_001170914.2  AHH85805.1  AHN49762.1  NP_991388.2  NP_991392.1  NP_001122147.2  ADX97523.2  ADR66025.1  AAW70378.1  NP_991389.1  NP_001025729.1  NM_001199335.1  HG514151.1  BAG55077.1 |
| *Danio rerio* | TLR3 | AAI07956.1 |
| *Ctenopharyngodon idella* | TLR3 | ABI64155.1 |
| *Cyprinus carpio* | TLR3 | ABL11473.1 |
| *Mus musculus* | TLR1 | [NP_001263374.1](https://www.ncbi.nlm.nih.gov/protein/NP_001263374.1?report=genbank&log$=prottop&blast_rank=1&RID=4UUEAU4Y013) |
| *Mus musculus* | TLR2 | AAH14693.1 |
| *Mus musculus* | TLR3 | AAH99937.1 |
| *Mus musculus* | TLR4 | AAF04278.1 |
| *Mus musculus* | TLR5 | NP_058624.3 |
| *Mus musculus* | TLR6 | AAH55366.1 |
| *Mus musculus* | TLR7 | AGX25544.1 |
| *Mus musculus* | TLR8 | AAK62677.1 |
| *Mus musculus* | TLR9 | NP_112455.2 |
| *Oncorhynchus mykiss* | TLR7 | XP_021453688.2 |
| *Salmo salar* | TLR8 | NP_001155165.1 |
| *Salmo salar* | TLR9 | NP_001117125.1 |
| *Salmo salar* | TLR18 | CDK60413.1 |
| *Paralichthys olivaceus* | TLR14 | AB576805.1 |
| *Trachinotus ovatus* | TLR14 | MH719083.1 |
| *Takifugu rubripes* | TLR7 | AAW69375.1 |
| *Takifugu rubripes* | TLR8 | AAW69376.1 |
| *Takifugu rubripes* | TLR9 | AAW69377.1 |
| *Takifugu rubripes* | TLR14 | AC156431.1 |
| *Callorhinchus milii* | TLR27 | XP_007893881.1 |
| *Ctenopharyngodon idella* | TLR8 | ADU33871.1 |
| *Ctenopharyngodon idella* | TLR18 | AIB55030.1 |
| *Cyprinus carpio* | TLR1 | BAU98379.1 |
| *Cyprinus carpio* | TLR2 | BAU98380.1 |
| *Cyprinus carpio* | TLR4 | BAU98382.1 |
| *Cyprinus carpio* | TLR5 | AGH15501.1 |
| *Cyprinus carpio* | TLR7 | BAJ19518.1 |
| *Cyprinus carpio* | TLR8 | AXL65589.1 |
| *Cyprinus carpio* | TLR18 | ATW66458.1 |
| *Cyprinus carpio* | TLR19 | MW411431 |
| *Danio rerio* | TLR1 | NP_001124065.1 |
| *Danio rerio* | TLR2 | NP_997977.1 |
| *Danio rerio* | TLR4ba | NP_001124523.1 |
| *Danio rerio* | TLR4bb | NP_997978.2 |
| *Danio rerio* | TLR5 | XP_001919052.2 |
| *Danio rerio* | TLR5b | NP_001124067.2 |
| *Danio rerio* | TLR8 | XP_002665954.4 |
| *Danio rerio* | TLR9 | NP_001124066.1 |
| *Danio rerio* | TLR18 | NP_001082819.1 |
| *Gallus gallus* | TLR1a | ACR26538.1 |
| *Gallus gallus* | TLR1b | ACR26464.1 |
| *Gallus gallus* | TLR2a | ACR26421.1 |
| *Gallus gallus* | TLR2b | ACR26376.1 |
| *Gallus gallus* | TLR3 | ABL74502.1 |
| *Gallus gallus* | TLR4 | NP_001025864.1 |
| *Gallus gallus* | TLR5 | [AFV92631.1](https://www.ncbi.nlm.nih.gov/protein/AFV92631.1?report=genbank&log$=prottop&blast_rank=1&RID=50RED027016) |
| *Gallus lafayetii* | TLR7 | ACR26207.1 |
| *Homo sapiens* | TLR1 | [AAC34137.1](https://www.ncbi.nlm.nih.gov/protein/AAC34137.1?report=genbank&log$=prottop&blast_rank=1&RID=50MYBTF101R) |
| *Homo sapiens* | TLR2 | [NP_001305716.1](https://www.ncbi.nlm.nih.gov/protein/NP_001305716.1?report=genbank&log$=prottop&blast_rank=1&RID=50MXCBKG013) |
| *Homo sapiens* | TLR3 | [AAH94737.1](https://www.ncbi.nlm.nih.gov/protein/AAH94737.1?report=genbank&log$=prottop&blast_rank=1&RID=50MW7UJZ013) |
| *Homo sapiens* | TLR4 | [NP_612564.1](https://www.ncbi.nlm.nih.gov/protein/NP_612564.1?report=genbank&log$=prottop&blast_rank=1&RID=4YV6FS00016) |
| *Homo sapiens* | TLR5 | [NP_003259.2](https://www.ncbi.nlm.nih.gov/protein/NP_003259.2?report=genbank&log$=prottop&blast_rank=1&RID=4YV5PT98013) |
| *Homo sapiens* | TLR6 | [NP_006059.2](https://www.ncbi.nlm.nih.gov/protein/NP_006059.2?report=genbank&log$=prottop&blast_rank=1&RID=4YHXWCFG016) |
| *Homo sapiens* | TLR7 | [AAZ99026.1](https://www.ncbi.nlm.nih.gov/protein/AAZ99026.1?report=genbank&log$=prottop&blast_rank=1&RID=4YHX1TUU013) |
| *Homo sapiens* | TLR8 | [AAZ95439.1](https://www.ncbi.nlm.nih.gov/protein/AAZ95439.1?report=genbank&log$=prottop&blast_rank=1&RID=4YHWHPY7016) |
| *Homo sapiens* | TLR9 | [EAW65192.1](https://www.ncbi.nlm.nih.gov/protein/EAW65192.1?report=genbank&log$=prottop&blast_rank=1&RID=4YH724X0016) |
| *Ictalurus punctatus* | TLR7 | [AEI59670.1](https://www.ncbi.nlm.nih.gov/protein/AEI59670.1?report=genbank&log$=prottop&blast_rank=1&RID=4YH654KW016) |
| *Ictalurus punctatus* | TLR8 | [AEI59672.1](https://www.ncbi.nlm.nih.gov/protein/AEI59672.1?report=genbank&log$=prottop&blast_rank=1&RID=4YBC3S6F01R) |
| *Ictalurus punctatus* | TLR9 | [XP_017323797.1](https://www.ncbi.nlm.nih.gov/protein/XP_017323797.1?report=genbank&log$=prottop&blast_rank=1&RID=4YBBRWT901R) |
| *Ictalurus punctatus* | TLR18 | [AEI59674.1](https://www.ncbi.nlm.nih.gov/protein/AEI59674.1?report=genbank&log$=prottop&blast_rank=1&RID=4YBB3371016) |
| *Ictalurus punctatus* | TLR25 | [AEI59680.1](https://www.ncbi.nlm.nih.gov/protein/AEI59680.1?report=genbank&log$=prottop&blast_rank=1&RID=4YAT7XMG016) |
| *Larimichthys crocea* | TLR7 | [AGO28200.1](https://www.ncbi.nlm.nih.gov/protein/AGO28200.1?report=genbank&log$=prottop&blast_rank=1&RID=4YAMM003016) |
| *Larimichthys crocea* | TLR8 | [XP_010741343.3](https://www.ncbi.nlm.nih.gov/protein/XP_010741343.3?report=genbank&log$=prottop&blast_rank=1&RID=4YAKP0ZX016) |
| *Larimichthys crocea* | TLR9 | [ACF60624.1](https://www.ncbi.nlm.nih.gov/protein/ACF60624.1?report=genbank&log$=prottop&blast_rank=2&RID=4Y94VWA9016) |
| *Labeo rohita* | TLR3 | AFD97495.1 |
| *Labeo rohita* | TLR22 | AGW43270.1 |
