# Supplementary figures and images for: Identification and functional characterization of a fish-specific tlr19 in common carp (Cyprinus carpio L.) that recruits TRIF as an adaptor and induces ifn expression during the immune response
Source: Vet Res. 2021 Jun 15;52:88. doi: 10.1186/s13567-021-00957-3 (PMC8207781; doi:10.1186/s13567-021-00957-3)

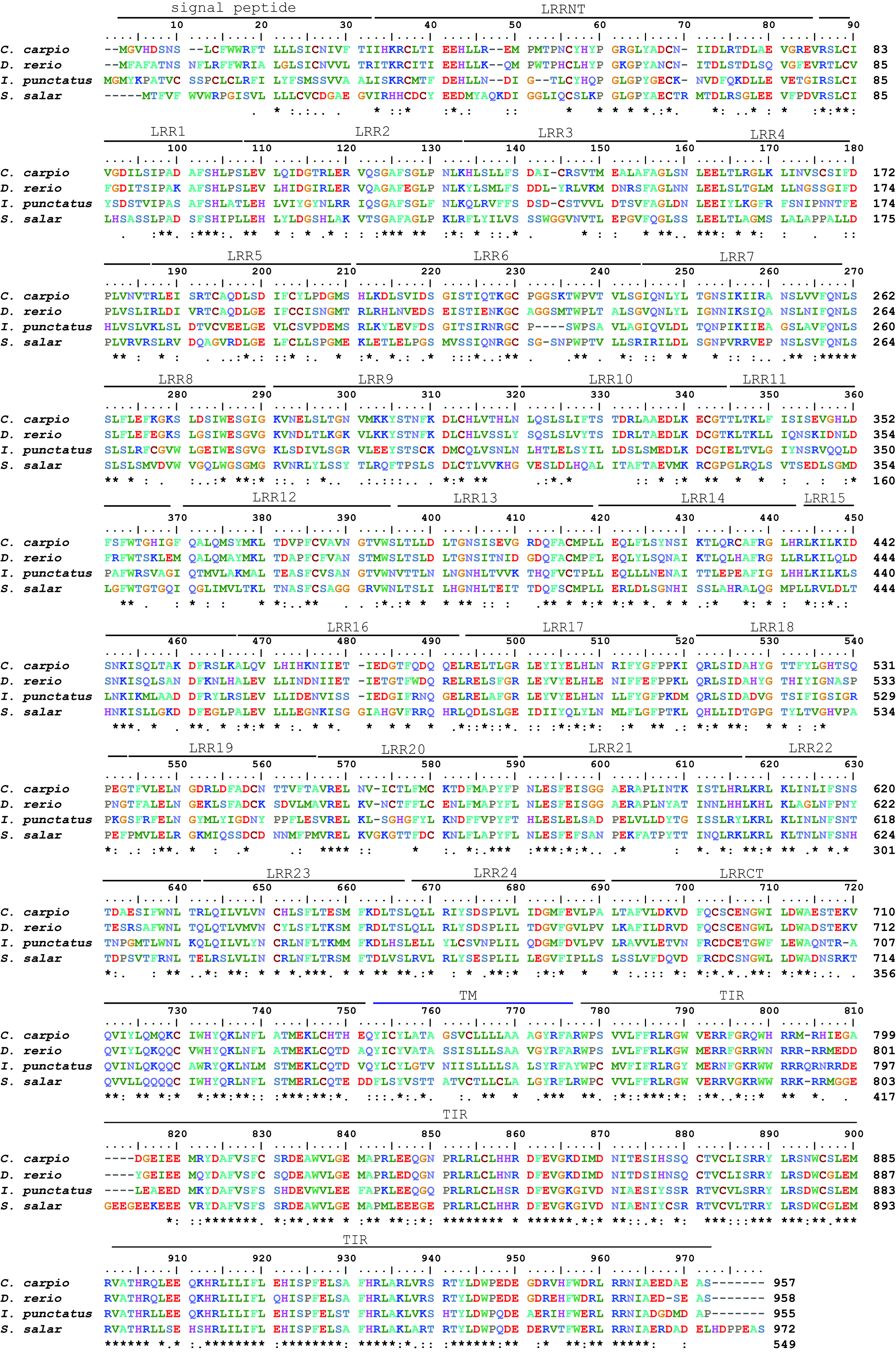

Supplement: Supplementary file 2 — Additional file 2. The multiple alignment analysis of CcTlr19. The sequences were aligned using the Clustal W method. Identical, conserved and similar substituted amino acid residues are indicated in (*), (: or .), respectively. [file 13567_2021_957_MOESM2_ESM.tif]

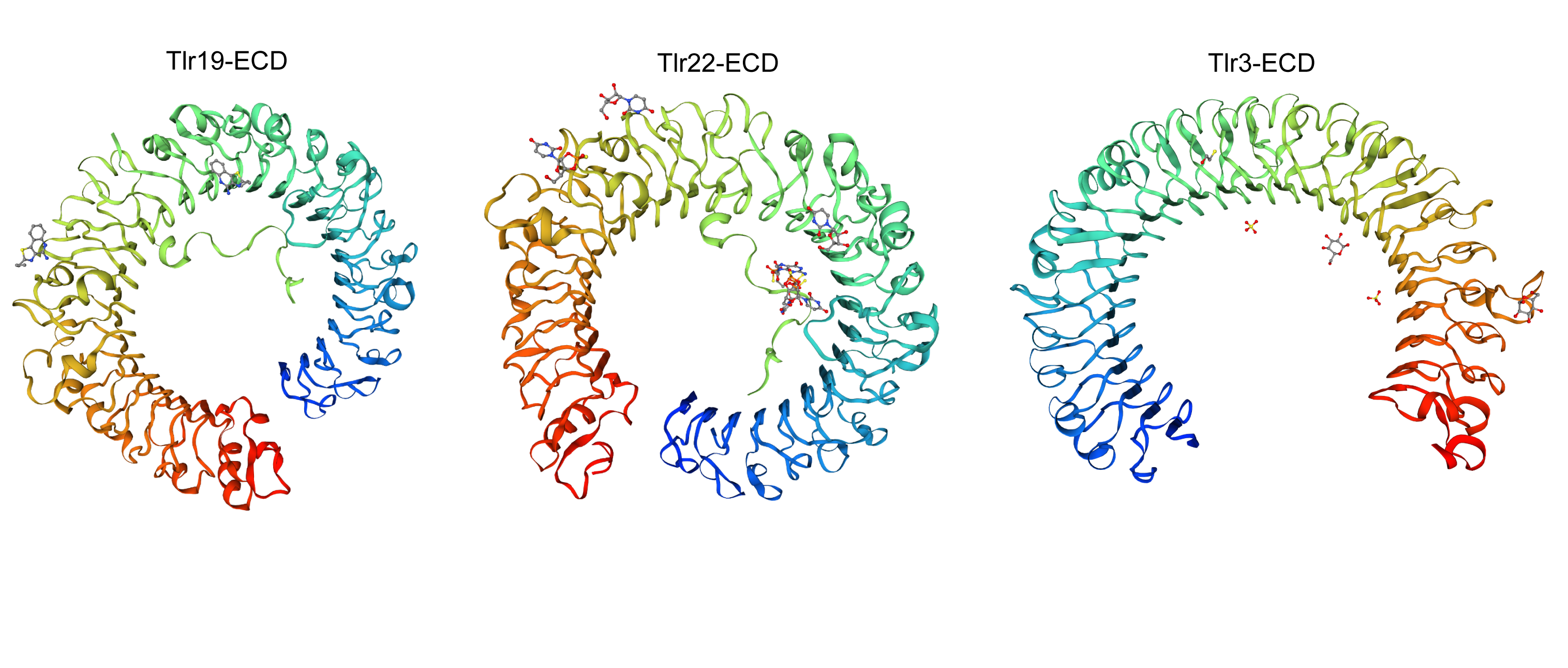

Supplement: Supplementary file 3 — Additional file 3. Modeled three-dimensional structure of CcTLR19, CcTlr3 and CcTlr22 ectodomain in a cartoon mode. [file 13567_2021_957_MOESM3_ESM.tif]
